# Treatment of isolated injuries of the posterior cruciate ligament—A 2025 Delphi‐based structured expert statement by the ligament injury committee of the German Knee Society

**DOI:** 10.1002/ksa.70187

**Published:** 2025-11-16

**Authors:** Christian Eberle, Danko Dan Milinkovic, Andrea Achtnich, Raymond Best, Philipp‐Johannes Braun, Lena Eggeling, Andree Ellermann, Martin Häner, Mirco Herbort, Jürgen Höher, Andreas Imhoff, Christoph Kittl, Julian Mehl, Natalie Mengis, Peter Müller, Daniel Niederer, Daniel Günther, Wolf Petersen, Thomas Pfeiffer, Sven Scheffler, Christian Schoepp, Thomas Stein, Thomas Stoffels, Amelie Stöhr, Tobias Gensior, Tobias Jung

**Affiliations:** ^1^ Arcus Sportklinik Pforzheim Germany; ^2^ Center for Musculoskeletal Surgery Charité‐University Medicine Berlin Germany; ^3^ Department for Orthopedic Sports Medicine Technical University Munich (TUM) Munich Germany; ^4^ Department of Orthopaedic and Sports Trauma Surgery Sportsclinic Stuttgart Stuttgart Germany; ^5^ Department of Sports Medicine University of Tuebingen Tuebingen Germany; ^6^ Department of Trauma and Orthopaedic Surgery BG Hospital Unfallkrankenhaus Berlin Germany; ^7^ Department of Trauma and Orthopaedic Surgery, Sports Traumatology BG Klinikum Hamburg Hamburg Germany; ^8^ Department of Orthopedics, Sportsclinic Berlin Martin Luther Hospital Berlin Germany; ^9^ OCM Clinic Munich Munich Germany; ^10^ Sportsclinic Cologne Köln Germany; ^11^ Department of Trauma, Hand and Reconstructive Surgery Westphalian Wilhelms University Muenster Muenster Germany; ^12^ Kantonsspital Baselland (Bruderholz) Liestal Switzerland; ^13^ Department of Orthopaedics and Trauma Surgery Musculoskeletal University Center Munich (MUM), University Hospital, LMU Munich Munich Germany; ^14^ SPORTHOLOGICUM® Frankfurt am Main, Center for Sport & Joint Injuries Frankfurt am Main Germany; ^15^ Institute of Occupational, Social and Environmental Medicine, Goethe University Frankfurt Frankfurt Germany; ^16^ Department of Orthopaedic Surgery, Trauma Surgery, and Sports Medicine, Cologne‐Merheim Medical Center (CMMC) Witten‐Herdecke University Cologne Germany; ^17^ Sporthopaedicum Berlin Berlin Germany; ^18^ Department of Arthroscopic Surgery, Sports Traumatology, and Sports Medicine BG Klinikum Duisburg gGmbH Duisburg Germany; ^19^ Department of Sports Medicine Goethe University Frankfurt Frankfurt am Main Germany; ^20^ OC Stadtmitte Berlin Germany; ^21^ Department of Trauma and Orthopaedic Surgery Schön Klinik Düsseldorf Düsseldorf Germany

**Keywords:** consensus, cruciate ligament, Delphi process, diagnostic, PCL, treatment

## Abstract

**Purpose:**

The main goal was to perform a modified Delphi process with the Ligament Injuries Committee of the German Knee Society (DKG) to structure and optimize the management of isolated posterior cruciate ligament (PCL) injuries.

**Methods:**

A structured modified Delphi approach was used to develop an expert statement. Steering group formulated an initial questionnaire and distributed it to 15 experienced knee surgeons (male/female 13/2, mean age 45 ± 5 years) of the working group in Round 1. Thirty‐one statements covering five thematic topics were then derived from the responses and comprehensive literature search (Medline, Scopus and Cochrane) using variations of different search terms (literature group). The statements underwent two rating cycles by the working group, using a 5‐point Likert scale in Round 2 and as a binary ‘agree/disagree’ in the final third round. Levels of evidence were assigned to each statement using standardized A–E and GRADE grading systems based on the available data.

**Results:**

High agreement (≥80%) was achieved for 24 of the 31 statements (range, 83%–100%), whereas for 7 agreement was <80% (range 63%–74%). The highest levels of agreement were reached for imaging modalities, treatment of PCL tibial avulsions, and preservation of native PCL fibres in reconstruction techniques, whereas the greatest divergence was observed regarding the role of leg axis and slope analyses and indications for corrective osteotomies, use of augmentation in reconstruction and post‐operative rehabilitation protocols. The available level of evidence across studies in the literature was predominantly low to moderate. Of the 31 statements, 17 were graded as expert opinion (E, GRADE: very low), 12 as case series (C; GRADE: low), and only 2 achieved higher levels of evidence (B2, GRADE: moderate).

**Conclusion:**

By providing structured treatment protocols, this Delphi‐based structured expert statement can support clinicians in day‐to‐day decision‐making and ultimately improve patient care and outcomes.

**Study Design:**

Expert survey.

**Level of Evidence:**

Level V.

**Abbreviations**
ACL: anterior cruciate ligament
DKG: Deutsche Kniegesellschaft (German Knee Society)
GRADE: Grading of Recommendations, Assessment, Development and Evaluation
PCL: posterior cruciate ligament
PCL‐R: posterior cruciate ligament reconstruction

## INTRODUCTION

Treatment algorithms for injuries of the anterior cruciate ligament (ACL) are well established in clinical practice [26, 58, 59]. Due to the significantly rarer occurrence of injuries of the posterior cruciate ligament (PCL) and the herewith associated limited availability of relevant data in the literature, the management of PCL injuries still presents a significant challenge for physicians [5, 8, 23, 36, 39, 54, 65, 67].

An isolated PCL injury is considered a partial or full tear of the PCL confirmed by clinical and radiological evaluation in the absence of concomitant ligamentous injuries (such as injuries to the ACL, medial and lateral collateral ligaments), posterolateral or posteromedial corner involvement [4, 8, 25, 31, 55]. In general, isolated PCL injuries are, depending on the rupture morphology, most commonly treated conservatively with favourable outcomes [33, 54, 57, 65, 79]. However, in cases of high‐grade instability in young, active, high‐risk populations, surgical reconstruction of the PCL may be indicated to restore joint stability and function and prevent potential sequelae [8, 14, 21, 48]. The anatomical complexity of the PCL and the inherent technical intricacies of surgical approaches make PCL injuries particularly difficult to manage surgically [2, 70, 79, 80, 81, 82]. Furthermore, a significant learning curve is associated with developing proficiency in performing surgical procedures to treat these injuries [2, 70, 79, 80, 81, 82].

The lack of clear standardized approaches in the available literature highlights the need for a clinical decision‐making guide. The primary objective of this study was thus to develop a comprehensive, expert‐driven treatment algorithm for managing isolated PCL injuries. A structured Delphi methodology, which enables iterative knowledge exchange between experts, facilitates the refinement of complex clinical questions and encourages converging of opinions through multiple rounds of structured feedback was used [51]. Additionally, the aim was to identify areas of significant divergence in expert opinion and highlight these gaps in the context of the existing literature. Ultimately, a treatment algorithm was proposed based on the analyzed data and comprehensive literature review.

## MATERIALS AND METHODS

The study was performed under the supervision of the Knee Ligament Committee of the German Knee Society (DKG). The expert panel consisted of 15 members of the DKG Ligament Injury Committee (male/female, 13/2; mean age 45 ± 5 years, mean years of experience 12 ± 3 years). All members were senior knee surgeons with extensive experience in the treatment of isolated and multi‐ligamentous knee injuries. Their professional focus and scientific contributions had positioned them as leading experts in Germany, ensuring that all participants in the process were well‐versed in the relevant pathology.

This Delphi‐based survey project followed a systematic approach as previously published [26]. A modified Delphi process, based on a standardized classic Delphi design, was employed to ensure controlled feedback and iterative refinement throughout the process [26]. The number of rounds was predefined as three, which is standard in Delphi methodology and consistent with prior publications [26].

A steering group was formed, consisting of the two senior authors (T.J. and C.E.), both experienced orthopaedic knee surgeons and long‐standing members of the Ligament Committee of the DKG. They were responsible for overseeing and coordinating the process, analyzing and incorporating the results of each round and providing feedback and final results. The group was selected based on clinical and academic expertise, and their role was limited to coordination and design.

Based on their clinical experience and comprehensive review of the literature, the steering group drafted an initial questionnaire, which was emailed to 15 expert panel members—comprising the ‘working group’ for general feedback. This initial survey allowed open‐ended responses and covered a wide range of questions regarding the management of isolated PCL injuries (i.e., indication, surgical and non‐operative treatment, diagnostic and post‐operative management); the responses were collected anonymously. The purpose of the initial questionnaire was to generate ideas and concepts related to the research question, taking into account the clinical experience and previous research of the panel members. A comprehensive literature review was then performed by the literature group (D.D.M. and D.G.) using major databases, including MEDLINE (via PubMed), Scopus and CENTRAL (Cochrane Library). Search terms included ‘posterior cruciate ligament’, ‘PCL’, ‘diagnosis’, ‘tibial slope’, ‘surgical treatment’, ‘conservative treatment’, ‘bracing’ and ‘outcome’, used in various combinations. The inclusion criteria were: (1) human studies, (2) English language, (3) publication years from 1980 to 2025 and (4) relevance to diagnosis or treatment of isolated PCL injuries. Animal and cadaveric studies were excluded from the evidence grading but reviewed for supplementary background data. Reference lists of selected articles were also screened to identify additional studies.

Based on the initial responses and the corresponding literature, 31 critical questions addressing five key topics: (a) indication and diagnostic; (b) operative‐; (c) non‐operative treatment; (d) post‐operative management and (e) outcome assessment, were formulated and then hypothetically answered by corresponding scientific statements by the steering group. These statements were carefully selected to effectively encompass all aspects of the aforementioned five major categories, under consideration of data from the available literature.

The corresponding level of scientific evidence was assigned to each statement based on the available literature and documented at the end of each statement, according to the A–E grading system outlined in Table 1, as published in previous consensus projects [18, 26]. In parallel, all included studies were systematically evaluated using the GRADE framework [11], which assesses the quality of evidence across four domains: risk of bias, inconsistency, indirectness, and imprecision. Based on this assessment, relevant studies were graded as high, moderate, low, or very low quality, and the overall grade was summarized for each statement.

**Table 1:** Explanation of the grading system for analyzed studies.

Grade	Type of Study
A1	Several (>2) level 1, randomized clinical studies with similar results or a meta‐analysis
A2	A single, level 1, randomized clinical trial
B	Prospective cohort study
B2	Any comparison group that is not level 1
C	Case series
D	Case report
E	Expert opinion/basic research

In the second step, the statements were then sent to all members of the working group, who then rated the statements using a 5‐point Likert scale, ranging from ‘I fully agree’ to ‘I fully disagree’ with an option for ‘I can't judge’. Free‐text comments were also encouraged to explain ratings or to express disagreement with the formulation or relevance of statements. The statements were further adapted based on the analysis of the responses, and a subsequent version was prepared for the third stage.

Before the final round of rating, a structured part in‐person and part online videoconference was held with all 15 panel members of the working group and two members of the steering group within the annual meeting of the DKG Ligament Committee. This open discussion allowed for clarification of ambiguous statements, review of the previous round's results, and further refinements. The meeting served to address remaining uncertainties and improve transparency, partially compensating for the absence of earlier live interactions.

The revised draft was then returned to the panel members for final round of assessment, requiring a simple dichotomization into ‘I agree’ or ‘I disagree’ with the statement, with no provision for additional comments.

### Data and statistical analysis

An agreement of 80% or above was considered a high level of agreement. Statements with less than 80% agreement were retained in the final report, and comments from earlier rounds were reviewed to assess reasons for lower levels, but no post hoc changes were made to the wording or inclusion of statements. The final Manuscript was approved by all members of the panel prior to final submission. Throughout this modified Delphi process, feedback was shared on the overall responses of all committee members [51].

Questionnaires were distributed using SoSci Survey (SoSci Survey GmbH), which enabled automatic tracking of completion. Data were processed using SPSS (version 20.0; IBM) and Excel 2019 (Microsoft). The overall percentage agreement among members of the expert panel was selected as the primary statistical outcome.

## RESULTS

All 15 members of the ‘working group’ participated in all three rounds of the Delphi process, with a 100% response rate in each round. The two members of the ‘steering group’ did not participate in the voting. No additional recruitment occurred, and there were no dropouts during the process. A total of 31 statements were assessed.

In total, 24 out of 31 statements reached high level of agreement (agreement ≥ 80%, range: 83%–100%), out of which 15 statements had a 100% agreement. Seven statements had a <80% agreement (range 63%–74%). This finding is particularly noteworthy, further highlighting the complexity and frequent divergence of opinion—even among leading experts—within the broad spectrum of isolated PCL injury management.

The majority of statements were based on low to very low quality of evidence, as reflected by 17 statements graded E (corresponding to GRADE = Very Low), 2 graded C (GRADE = Low) and only two graded B2 (GRADE = Moderate). It should be noted that these ratings refer strictly to the level of available evidence, not to the strength of recommendations.

**Figure 1:**
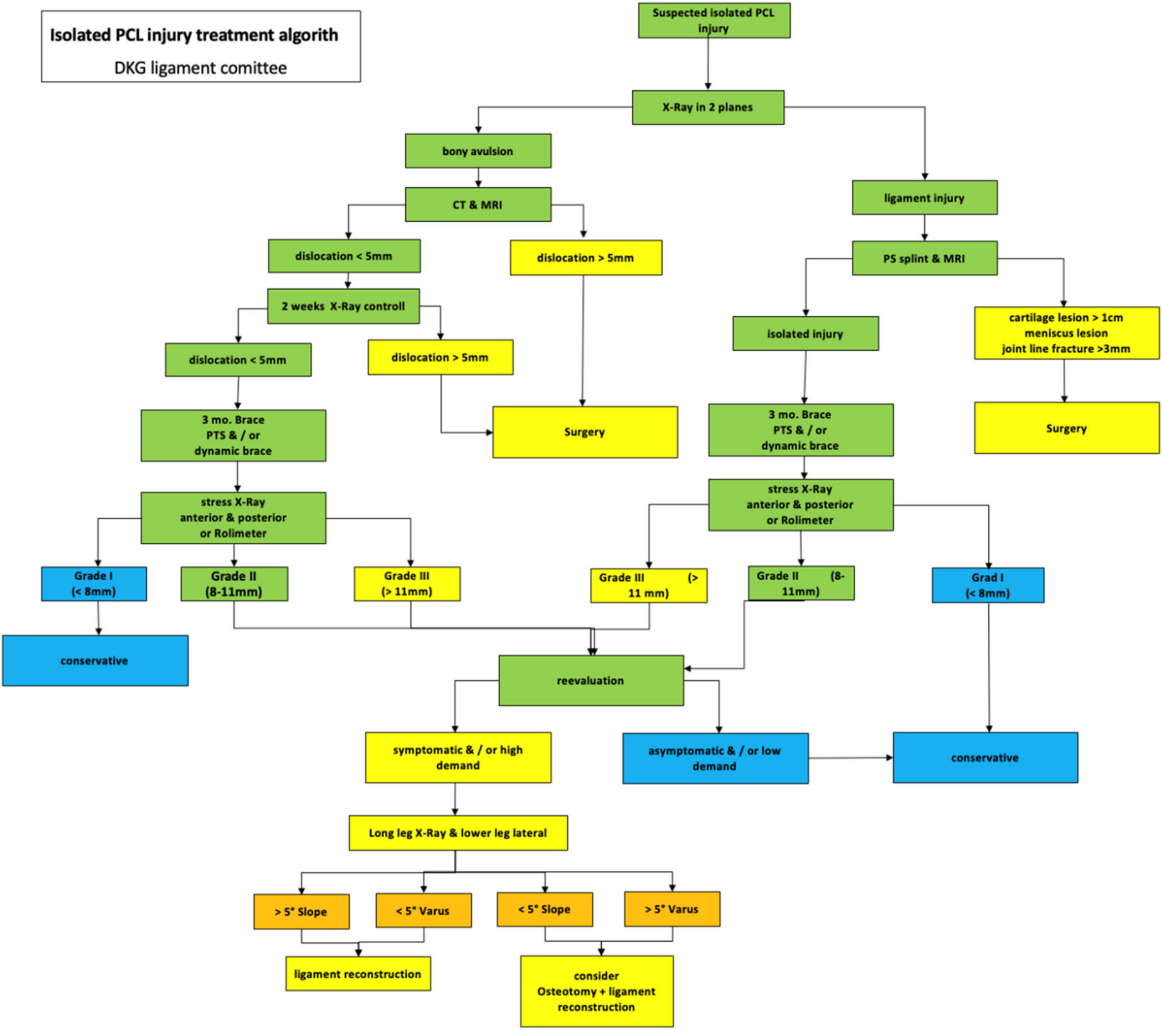
Treatment of isolated PCL injuries algorithm. CT, computed tomography; MRI, magnetic resonance imaging; PCL, posterior cruciate ligament; PTS, posterior translation stabilizing.

Based on the results of the survey and comprehensive review of the available literature, the Ligament Committee of the DKG has proposed a treatment algorithm presented in Figure 1.

Summary of the questions and corresponding statements for the five subject topics:

### Topic 1: Indication and diagnostic

***Q1***. ***How should an isolated tibial PCL avulsion injury be treated?***

Experts emphasize precise surgical criteria in the treatment of tibial avulsion injuries, foremost taking into account the degree of displacement of the bony fragment [17]. Preference should be given to surgically treating the more severely displaced avulsion injuries with a threshold of bony fragment displacement greater than 5–6 mm [17, 23, 82] (**Grade of Evidence**: *C; GRADE: low;* **Vote**: *90% agreement*).

***Q2***. ***Does the decision on the management of an acute isolated PCL rupture depend on the location of the ligament rupture?***

In the absence of clear, comprehensive data, the majority of experts agree that the location of rupture in acute isolated PCL injuries is not the sole determining factor in treatment decisions [4, 37, 39, 54, 57, 65, 67] (***Grade of Evidence***: *C*; *GRADE: low;* **Vote**: *80% agreement*).

***Q3. Does the decision on the management of an acute isolated PCL rupture depend on the morphology of the ligament rupture (partial vs. complete)?***

There is broad support among experts for conservative management in cases of both isolated incomplete and complete ruptures at baseline, which correlates with studies showing a high healing rate of the ligament, regardless of the rupture grade [39, 44, 54, 65, 73, 81]. Partial rupture of the PCL should be considered with less than 7 mm of antero‐posterior tibial translation on stress radiographs, and greater than 7 mm of residual ligament thickness on MRIs [39, 44, 54, 65, 73, 81] (***Grade of Evidence***: *C*; *GRADE*: *low*; **Vote**: *100% agreement*).

***Q4. Should radiological analysis of the leg axis and tibial slope always be performed when considering treatment of acute isolated PCL injuries?***

Low agreement was not reached for this question, with 60% of experts agreeing that leg axis analysis should be incorporated into the treatment decision‐making algorithm for acute isolated PCL injuries. In the absence of supportive data, this disagreement among experts is understandable. However, the general recommendation is that the need for radiographic assessment of leg alignment and tibial slope in acute isolated PCL rupture should primarily be based on clinical suspicion of significant coronal deformities that may contribute to instability and/or impaired joint function, assessed on a case‐by‐case basis [15, 16, 52] (**Grade of Evidence**: *E; GRADE: very low;* **Vote**: *60% agreement*).

***Q5. Should radiographic analysis of the leg axis and tibial slope be performed after failed conservative treatment with persistent posterior translational instability in cases of isolated PCL injuries?***

There is strong agreement among experts that radiographic analysis of the leg axis and tibial slope should form part of a comprehensive diagnostic workup to identify potential confounding factors once conservative treatment has failed [15, 49] (**Grade of Evidence**: *E; GRADE: very low*; **Vote**: *100% agreement*).

***Q6. Should the treatment consideration be influenced by the results of leg axis and slope analysis in cases of acute isolated PCL rupture?***

The experts advise that both the coronal profile of the leg (when clinical suspicion is evident) and the tibial slope should be considered, as the decision to pursue conservative treatment should be more critically evaluated in the presence of severe pathoanatomical predisposing factors [78, 79, 81] (**Grade of Evidence**: *E; GRADE: very low;* **Vote**: *100% agreement*).

***Q7. Should a varus/valgus‐producing osteotomy be performed to correct the corresponding extra‐articular deformity in cases of acute isolated PCL rupture with a coronal axis deviation greater than 3°?***

The expert group does not recommend corrective osteotomy based solely on a coronal axis deviation greater than 3° as a definitive benchmark. Instead, they recommend that the indication for surgery should be primarily based on clinical symptoms related to the deformity and significant functional joint impairment [13, 15, 43, 52, 71] (**Grade of Evidence**: *E*; *GRADE: very low;* **Vote**: *100% agreement*).

***Q8. Should a proximal flexion tibial osteotomy be performed in the acute setting for an abnormally reduced posterior tibial slope (<5°) in cases of acute isolated PCL rupture?***

In the absence of comprehensive evidence, the expert panel advises against proximal flexion tibial slope–correction osteotomy in acute cases of isolated PCL injury [13, 15, 43, 49, 71, 80] (**Grade of Evidence**: *E*; *GRADE*: *very low*; **Vote**: *100% agreement*).

***Q9. Should a proximal flexion tibial osteotomy be performed in cases of chronic PCL insufficiency with a reduced posterior tibial slope (<5°)?***

High agreement was not reached among experts for this question, with 60% agreeing that a proximal flexion tibial osteotomy should be considered in chronic PCL insufficiency when the posterior tibial slope is reduced (<5°). The variation in expert opinion reflects differing interpretations of the biomechanical effectiveness of slope‐correcting osteotomies in restoring posterior stability in chronic cases [13, 43, 52, 54, 67, 78, 79] (**Grade of Evidence**: *C; GRADE: low*; **Vote**: *60% agreement*).

***Q10. In case of a posterior tibial slope*** < ***5° after failure of conservative treatment, should a flexion tibial osteotomy combined with PCL‐R be performed?***

High agreement was not reached for this question, with 70% of experts agreeing that, in cases with a posterior tibial slope <5° and failed conservative treatment, a combined flexion tibial osteotomy and PCL‐R may be indicated depending on individual patient factors. The general recommendation is to approach such cases with caution and to base the decision on the specific pathoanatomical profile and clinical stability [13, 43, 52, 54, 67, 78, 79] (**Grade of Evidence**: *C*; *GRADE*: *low*; **Vote**: *70% agreement*).

***Q11. In case of a posterior tibial slope*** < ***5°, should flexion tibial osteotomy be performed as the isolated treatment after failure of conservative management*** **?**

Full agreement was not reached for this question, with only 60% of experts agreeing that a flexion tibial osteotomy could be considered as an isolated treatment option in cases of posterior tibial slope <5° after failed conservative management. The divided opinion reflects uncertainty about the effectiveness of slope‐correcting osteotomy as a stand‐alone procedure, which is consistent with the current literature showing limited evidence and a lack of clearly defined indication criteria [13, 15, 16, 49, 52, 53, 80] (**Grade of Evidence**: *E*; *GRADE: very low*; **Vote**: *60% agreement*).

***Q12***. ***Should stress radiographs always be obtained following isolated PCL injuries?***

The agreement among panel members emphasizes the importance of side‐to‐side comparisons, supported by studies that highlight their role in assessing joint translational stability [27, 30, 32, 35, 37, 55, 76]. Radiographs should always be performed in both the antero‐posterior and poster‐anterior direction for a complete assessment of ligamentous stability (**Grade of Evidence**: *C*; *GRADE: low;* **Vote**: *100%*). The expert group believes that the timing of radiographs should be based on clinical milestones and the degree of instability, rather than on a fixed schedule. The general recommendation is to avoid imaging immediately after injury, whereas the decision to perform radiographs at 6 weeks and/or 3 months post‐injury should be based on clinical assessment of joint stability on an individual basis (**Grade of Evidence**: E; GRADE: very low; **Vote**: 80% *agreement*).

### Topic 2: Non‐operative treatment

1. ***Should a shorter period of immobilization (<6 weeks) be used in conservative treatment of isolated tibial PCL avulsion injuries than for ligamentous PCL injuries?***

High agreement was not reached for this question, with 60% of experts agreeing that a shorter period of immobilization, <6 weeks, should be used in the conservative treatment of isolated tibial PCL avulsion injuries compared to ligamentous PCL injuries. Several treatment regimens have been reported, with no evidence to support one as superior to the others [17, 23, 82], which is reflected in the split in expert opinion as well. The general approach seems to reflect the opinion that, while shorter immobilization is associated with better early mobilization, longer immobilization may be associated with more secure healing and less residual instability [33, 41, 75] (**Grade of Evidence**: *E; GRADE: very low;* **Vote**: *60% agreement*).

2. ***Should follow‐up radiographs be regularly performed during the course of conservative treatment to monitor the success of isolated PCL avulsion injury management?***

Experts recommend regular radiographic imaging beginning as early as two to three weeks after injury to monitor healing progress, enable early detection of secondary dislocations, and allow timely adjustment of treatment if necessary [17, 23, 33, 41, 75] (**Grade of Evidence**: *C; GRADE: low;* **Vote**: *100% agreement*).

3. ***Which types of braces (static, dynamic functional and/or non‐dynamic functional) and for what duration of time should be used in the conservative management of acute isolated PCL injuries?***

Most experts support the use of a posterior tendon‐stabilizing static brace for the first six weeks post‐injury, which is consistent with evidence that prolonged static stabilization bracing promotes ligament healing in the early phases of recovery [33, 41, 75] (**Grade of Evidence**: *C; GRADE: low;* **Vote**: *80% agreement*). Experts recommend a combination of static and dynamic braces for initial stability and post‐acute mobility: static braces are preferred for up to six weeks, followed by dynamic braces for the next six weeks, although the low level of evidence leaves the decision largely to the treating clinician [3, 33, 56, 65, 67, 73, 75] (**Grade of Evidence**: *E; GRADE: very low;* **Vote**: *80% agreement*). The experts agree that the non‐dynamic braces should not be used as the sole means of immobilization for acute isolated PCL ruptures, as it is believed that non‐dynamic orthoses alone do not provide sufficient support for effective PCL rehabilitation [31, 75] (**Grade of Evidence**: *E; GRADE: very low;* **Vote**: *100% agreement*).

4. ***Should the duration of brace treatment for isolated PCL injuries not be less than three months?***

Strong support for a treatment period of at least three months underscores the importance of prolonged orthotic support, consistent with guidelines recommending extended use to optimize outcomes [60, 67, 73, 75, 79] (**Grade of Evidence**: *E; GRADE: very low;* **Vote**: *80% agreement*).

### Topic 3: Operative treatment

1. ***Are open growth plates a contraindication to surgical treatment of PCL injuries in skeletally immature patient population?***

The experts agree that open growth plates are not a contraindication and strongly support surgical treatment of isolated PCL injuries in skeletally immature patients [21, 34, 71, 74, 81] (**Grade of Evidence**: *E; GRADE: very low;* **Vote**: *100% agreement)*.

2. ***What concomitant injuries should be addressed during the primary surgical procedure in acute isolated PCL ruptures?***

The expert panel emphasizes the need for treatment of repairable meniscal lesions at the earliest possible time after injury [19, 54] (**Grade of Evidence**: *B2, GRADE: moderate;* **Vote**: *100% agreement*). Furthermore, the panel recommends that all acute traumatic grade III–IV° (ICRS) cartilage injuries larger than 1 cm² and fractures of the articular surface with an impression larger than 2 mm in the weight‐bearing cartilaginous zones should also be treated with primary surgery [4, 19, 38, 57, 62, 65, 82] (**Grade of Evidence**: *C; GRADE: low;* **Vote**: *100% agreement*).

3. ***Should internal ligament bracing surgical technique be used for acute isolated PCL ruptures?***

There is strong agreement supporting the use of internal bracing for acute PCL injuries requiring treatment in a relatively acute setting (e.g., time‐sensitive meniscal repairs) [20, 45, 63, 70, 72, 79, 81, 82] (**Grade of Evidence**: *C; GRADE: low;* **Vote**: *100% agreement*).

4. ***Does the indication for internal ligament bracing technique in acute isolated PCL ruptures, depend on the location of the ligament rupture?***

The experts strongly recommend the use of internal bracing technique in cases of proximal (femoral) and distal (tibial) PCL ruptures, supported by studies showing positive functional outcomes following bracing techniques when this localization of ligament rupture is present [23, 29, 33, 67, 81] (**Grade of Evidence**: *C; GRADE: low;* **Vote**: *80% agreement*).

5. ***Which tendon autograft options should be used routinely for primary PCL‐R in isolated PCL injuries?***

Hamstring grafts are strongly supported among experts as the primary autograft option for isolated PCL‐R, with extensive literature demonstrating their effectiveness in providing favourable outcomes [23, 39, 67] (*Evidence grade B2, GRADE: moderate; agreement, 100%*). Quadriceps tendon autografts are still largely reserved for revision cases but also represent a viable and biomechanically sound option for primary reconstruction [2, 23, 44] (**Grade of Evidence**: *E, GRADE: very low;* **Vote**: *100% agreement*).

6. ***Should primary PCL‐R always be performed with additional suture/tape augmentation as a ligament brace?***

Full agreement was not reached for this question, with 60% of experts agreeing that additional suture or tape augmentation (ligament bracing) should not be used routinely but rather be considered on a case‐by‐case basis. In the absence of clear evidence, the general recommendation is that the decision to use augmentation material should depend on the rupture pattern and location, joint morphology, footprint anatomy, and the surgeon's experience and preference [20, 29, 79] (**Grade of Evidence**: *E; GRADE: very low;* **Vote:** *60% agreement*).

7. ***Should primary PCL‐R always be performed with preservation of remaining native PCL fibres whenever possible*** **?**

Unanimous agreement exists between experts highlighting the value of preserving native structures, supported by evidence of improved healing once the remaining stump is preserved [23, 60, 78, 79] (**Grade of Evidence**: *C; GRADE: low;* **Vote:** *100% agreement*).

### Topic 4: Post‐operative management

1. ***Should rehabilitation protocols following internal bracing be different from those following PCL‐R with tendon graft options?***

High agreement was not reached for this question, with 70% of experts agreeing that rehabilitation protocols following internal bracing and tendon graft reconstruction should differ. The variation in expert opinion is consistent with differing approaches reported in the literature [5, 60, 68, 79]. Nonetheless, the general recommendation is that physical therapy should begin as early as possible, with progression based on individual patient characteristics, healing progression, and both clinical and subjective stability (**Grade of Evidence**: *E; GRADE: very low;* **Vote:** *70% agreement)*.

### Topic 5: Outcome assessment

1. ***Is the assessment of joint posterior stability after isolated PCL therapy using the Rolimeter and/or KT‐1000 (conservative and/or surgical) alone considered inadequate?***

Experts believe that neither the Rolimeter nor the KT‐1000 should be used as a sole assessment tool and should be combined with clinical examination and stress radiographs as part of a comprehensive assessment (**Grade of Evidence**: *E; GRADE: very low;* **Vote**: *80% agreement*).

*2*. ***Should the outcome of conservative therapy be assessed with stress radiographs at least 3 months after the start of therapy in the treatment of isolated PCL injuries*** **?**

In the absence of consistent evidence, the majority of experts recognize the importance of radiographic monitoring to assess treatment efficacy and recommend side‐to‐side stress radiographs at the 3‐month milestone [32, 65, 67, 79] (**Grade of Evidence**: *E; GRADE*: *very low;* **Vote**: *80% agreement*).

## DISCUSSION

The key finding of this survey project was that there is strong agreement among the expert panel on the majority of key aspects of the management of isolated PCL injuries. However, certain areas remain controversial, with differing opinions reflected in the current evidence base. In the absence of clear guidelines, this expert statement and proposed treatment algorithm (Figure 1) provides a valuable framework for optimizing the management of isolated PCL injuries.

Depending on the rupture morphology, non‐operative management remains the first‐line approach for acute isolated PCL injuries due to the self‐healing potential of the ligament [1, 4, 8, 40, 55]. Our expert panel statement in favour of non‐surgical approaches is in agreement with long‐term studies which have consistently demonstrated favourable clinical and functional outcomes, particularly for Grade I and II PCL injuries and total proximal PCL tears [4, 12, 40, 48, 54, 55, 64, 67, 79].

However, there was a high agreement among the panel experts that non‐operative management may not be sufficient, particularly in a young, active, high‐risk patient population with clinical high‐grade instability. In these cases, surgery is usually indicated to restore stability and prevent long‐term sequelae [2, 4, 14, 21, 28, 77]. Advances in surgical techniques have greatly expanded the treatment options for PCL injuries. Options such as single‐bundle and double‐bundle reconstructions using transtibial or tibial inlay and onlay techniques can be performed in open, arthroscopically assisted, or fully arthroscopic procedures, each offering specific advantages and challenges [19, 55, 65, 79]. There was a general panel agreement of timely interventions to address meniscal and cartilage injuries in combination with various PCL internal bracing or reconstruction techniques, also fitting the corresponding literature [8, 19, 36, 38, 62, 65].

For PCL tibial avulsions, surgical reduction is generally recommended for displacements larger than 5–6 mm, with current literature favouring minimally invasive techniques due to their precision and reduced morbidity [8, 17, 34, 79, 82]. This agreement is in line with the literature, which advocates a precise anatomical surgical technique to reduce the bone fragment [8, 17, 34, 79, 82]. However, there is disagreement regarding the duration of post‐operative immobilization following these surgical procedures in line with the absence of clear guidelines in the literature, with some supporting shorter immobilization to promote early mobilization and others recommending longer immobilization to achieve adequate osseous healing [17, 34, 66, 69]. Furthermore, for complete or avulsion PCL injuries in skeletally immature patients, surgical intervention remains a key focus [34, 66, 69]. Studies emphasize that careful surgical techniques can effectively stabilize the knee without compromising growth potential or limb developmental alignment [21, 23, 34, 74].

Graft selection remains a critical component of surgical planning. Autografts, particularly hamstring grafts, are widely used due to their biological compatibility, while quadriceps tendon grafts are still commonly preferred for revision surgery [2, 5, 9, 40, 42, 47, 68]. Allograft options, on the other hand, can reduce operative time and avoid donor site morbidity, but are associated with increased cost and are less available in this region [4, 8, 29, 65]. Although current evidence remains inconclusive regarding the complications related to the use of allografts in PCL‐R, several studies in ACL surgery have reported increased tunnel widening and osteolysis associated with the use of allografts, potentially due to limited biological integration and sterilization‐related graft degradation [10, 24]. Nevertheless, current evidence suggests comparable outcomes with different graft types, highlighting the importance of tailoring graft selection to the specific case and preference and expertise of the surgeon [42, 46, 47, 61].

Internal ligament bracing presents a viable technical option as well, with recent studies suggesting that it can provide improved tibial translational stability and supports the healing potential of the PCL, particularly when used as a primary augmentation or alongside autograft reconstructions [6, 50, 81]. Experts recognize internal bracing as a valid surgical option, particularly for acute tears with concomitant injuries requiring timely interventions, as this technique minimizes donor site morbidity, allows smaller tunnel sizes and preserves native tissue integrity [6, 50, 81]. However, long‐term data and higher‐quality studies are needed to further validate its efficacy and address concerns such as potential reaction to synthetic materials [17, 65, 77, 79].

Decreased tibial slope correlates with increased posterior tibial translation, placing significantly more stress on the PCL and potentially leading to higher rates of re‐injury and graft failure [49, 53, 71, 78, 80]. In recent years, tibial flexion osteotomy has emerged as a viable strategy to correct significant slope deformities, particularly in chronic insufficiency and revision cases with promising outcomes [13, 15, 52]. However, studies are inconclusive as to when (combined or prior to the index, or only revision surgery) and, importantly, at what threshold (i.e., degree of slope) a decreased tibial slope should be corrected. These too are among the main points of disagreement between the expert panel members, as well. While the evidence base for slope correction in cases of PCL injuries is still evolving, evidence from ACL research suggests that correcting increased tibial slope improves force distribution and reduces graft stress, potentially paving the way for similar advances in PCL management.

The panel members strongly recommended stress radiographs, as an essential diagnostic tool, facilitating side‐by‐side comparisons crucial for early work‐up and treatment monitoring [22, 27, 30, 32, 35, 60, 67, 76]. Although recommendations on the timing of radiographic assessments vary, experts suggest routine imaging at the 3‐month milestone in all cases, while earlier—at 6‐week post‐injury milestone assessments—should be reserved for specific clinical indications to identify potential complications and allow timely interventions [22, 27, 30, 36].

The experts' panel supports the use of static stabilizing orthoses within the first 6 weeks post‐injury, consistent with studies suggesting that prolonged stabilization promotes ligament healing [7, 37, 55, 64, 73, 79]. However, there was a lower agreement level on the role of dynamic orthoses in the acute phase, highlighting the variability in clinical practice and the ongoing debate about their effectiveness in providing adequate stability in the initial stages of treatment [33, 41, 75]. Although evidence is limited, most experts believe that the combined approach with the use of static in the acute (up to 6 weeks) and dynamic braces in post‐acute phases (up to 3 months post‐injury) may offer a balance between stability and preserving mobility of the joint.

The experts emphasize the importance of careful assessment of the individual patient's risk factor profile initially and at different stages of follow‐up, as shown in the treatment algorithm presented in Figure 1. Accordingly, the clinical and radiological evaluation of each individual case should include an assessment of the type of injury, degree of instability, progression or compensation at the index and follow‐up visits, but also take into account the various demographic factors and the individual's level of activity and subjective demands. This will allow the most appropriate management strategy and timing to be determined specifically for each patient, with the possibility of modifying the approach at any time based on the above contributing factors. Furthermore, from a socio‐economic perspective, the introduction of more standardized diagnostic and treatment guidelines can help to reduce over‐ and/or misdiagnosis, as well as under‐ or overtreatment, thereby reducing the overall cost of individual treatments in the future.

### Limitations

There are certain limitations that should be considered. Namely, the online nature of the process meant that the panel members were geographically dispersed, and most communication took place within the structured framework of the online questionnaire. However, this limitation can also be seen as an advantage as it helped to minimize the potential bias of dominant individuals influencing the decision‐making process. The limited number of participants (*n* = 15) and members of the expert panel belonging to a single region in Western Europe can be seen as limitation in terms of generalizability as well. Another limitation of this study is that the panel consisted only of trauma and orthopedic surgeons, without the inclusion of professionals who primarily advocate non‐operative treatment. The absence of certain perspectives may have introduced a slight bias towards operative management. However, the nature of the questions suggests that responses were fairly balanced between operative and non‐operative approaches, with clearly defined thresholds and indicators considered for surgical intervention. Furthermore, the use of a binary agreement format in the final round may have oversimplified complex clinical opinions and could have led to less agreement among the experts. However, we strongly believe that initially allowing free‐text comments, continuous feedback between rounds and the meeting held before the final round of voting have minimized this factor. Finally, a gender disparity was noted within the panel, with a predominance of male participants. This may limit the representativeness of the group and should be acknowledged as a potential source of bias.

## CONCLUSION

By providing structured treatment protocols, this Delphi‐based structured expert statement can support clinicians in day‐to‐day decision‐making and ultimately improve patient care and outcomes. In addition, outlining the areas with the least agreement among experienced high‐volume experts—and the lack of supporting literature—highlights the topics that warrant further investigation in the future.

## AUTHOR CONTRIBUTIONS

All authors have made substantial contributions to the conception and design, acquisition of data and analysis and interpretation of data.

## CONFLICTS OF INTEREST STATEMENT

Danko Dan Milinkovic and Christoph Kittl are members of the Editorial Board of the KSSTA. Mirco Herbort: Consultant: Medacta International, Arthrex, Stryker, Enovis and Royalties: Medacta International. Thomas Stoffels: Consultant: OPED. Wolf Petersen: Consultant: Karl Storz, Stryker, Geistlich and Arthrex. Julian Mehl: Arthrex and Enoviscos. Andreas Imhoff: Consultant: Arthrex, Arthrosurface/Anika Inc., Medi Bayreuth and Royalties: Arthrex and Arthrosurface/Anika Inc., Daniel Guenther: Consultant: Arthrex, Stryker, Karl Storz, Medical magnesium and Lecture fees: Geistlich, Codon, Rimasys, Medi, Anika, Telos and Travelling costs: Smith & Nephew. The remaining authors declare no conflicts of interest.

## ETHICS STATEMENT

The ethics statement is not available.

## Data Availability

Raw data can be made available upon request.
